# Pediatric premedication: a double-blind randomized trial of dexmedetomidine or ketamine alone versus a combination of dexmedetomidine and ketamine

**DOI:** 10.1186/s12871-017-0454-8

**Published:** 2017-11-29

**Authors:** Hui Qiao, Zhi Xie, Jie Jia

**Affiliations:** 1 Department of Anesthesiology, The Eye, Ear, Nose and Throat Hospital of Fudan University, 83 Fenyang Road, Shanghai, 200031 China; 2 Department of Anesthesiology, Shanghai Deji Hospital, Qingdao University, Shanghai, China

**Keywords:** premedication, dexmedetomidine, ketamine, pediatric patients

## Abstract

**Background:**

Preoperative anxiety is common in pediatric patients. When dexmedetomidine is used alone for sedation as premedication, children tend to awaken when separated from their parents, and body movements occur during invasive procedures. We tested the hypothesis that the combination of dexmedetomidine and ketamine may be a useful premedication to alleviate preoperative anxiety and improve cooperation during intravenous cannulation in pediatric patients, while producing minimal adverse events.

**Methods:**

A total of 135 children, aged 2–5 years and American Society of Anesthesiologists status I–II, scheduled for eye surgery were randomly allocated to receive intranasal dexmedetomidine 2.5 μg/kg (group D), oral ketamine 3 mg/kg and intranasal dexmedetomidine 2 μg/kg (group DK), or oral ketamine 6 mg/kg (group K) 30 min before surgery. Sedation state was evaluated every 10 min after premedication and emotional state was assessed during separation from their parents and peripheral intravenous cannulation. Adverse events were recorded for 24 h postoperatively. The primary endpoint was the rate of successful intravenous cannulation.

**Results:**

The rate of successful venous cannulation was 47% with dexmedetomidine alone, 68% with ketamine alone, and 80% with combined premedication (*P* = 0.006). The rate of satisfactory separation from parents was not different among groups. The incidence of adverse events was higher in group K compared with the other two groups (postoperative vomiting, *P* = 0.0041; respiratory-related complications during the perioperative period, *P* = 0.0032; and postoperative psychological/psychiatric adverse events, *P* = 0.0152).

**Conclusion:**

The combination of intranasal dexmedetomidine 2 μg/kg and oral ketamine 3 mg/kg produces satisfactory separation from parents and more successful venous cannulation, allowing children to smoothly accept induction of general anesthesia.

**Trial registration:**

Chinese Clinical Trial Register (Unique identifier: ChiCTR-TRC-14004475, Date of registration: 2 April 2014).

## Background

For pediatric patients undergoing general anesthesia, personality and behavior in the early postoperative period tend to be adversely affected if patients fail to cooperate before the induction of anesthesia [1]. Appropriate preoperative communication, being accompanied by their parents during anesthesia induction, medication interventions, and other methods are commonly used to lessen preoperative nervousness and anxiety in children. Medication interventions are widely used in clinical practice, and ideal premedication attributes include prompt onset of action, short duration, simple route of administration that is readily accepted by children, minimal side effects, reliable pain relief, and regulation of autonomic responses [2]. To date, there remains no widely-accepted premedication regimen.

Dexmedetomidine is an alpha 2-adrenoreceptor agonist that provides sedation, analgesia, and anxiolysis without respiratory depression in clinical practice. Its colorless and odorless properties make dexmedetomidine suitable for intranasal administration. Preoperative intranasal instillation of 1–2 μg/kg dexmedetomidine has been reported to produce sedation and ameliorate separation anxiety [3, 4].

Oral ketamine is commonly used as a premedication for pediatric patients. Oral administration of ketamine is characterized by a high first-pass elimination effect and potential undesirable postoperative side effects, including salivation, delirium, and anxiety [5].

Dexmedetomidine and ketamine exhibit complementary pharmacological effects. Dexmedetomidine reduces cardiovascular responses and postoperative psychiatric adverse reactions attributed to anesthesia induction with ketamine [6]. We conducted the current study to investigate whether the combination of dexmedetomidine and a low dose of ketamine in pediatric patients improved cooperation during invasive procedures (intravenous cannulation) and alleviated preoperative anxiety, while producing minimal adverse events.

## Methods

This trial was approved by the institutional review board of the Eye and ENT hospital affiliated with Fudan University and registered at the Chinese Clinical Trial Registry with registration number ChiCTR-TRC-14004475. The parents of all children in the study provided written, informed consent. A total of 135 children scheduled for eye surgery under general anesthesia, who were 2 to 6 years old with an American Society of Anesthesiologists’ status of I–II and a body mass index of 15–18 kg/m^2^, were enrolled in the study. The exclusion criteria included a recent respiratory tract infection, mental disorder, severe dysfunction of the central nervous system, increased intracranial pressure, or a history of dexmedetomidine allergy. Using a simple randomization procedure with computer-generated allocation, patients were randomly assigned to 1 of 3 treatment groups: group D (intranasal dexmedetomidine 2.5 μg/kg), group DK (intranasal dexmedetomidine 2 μg/kg and oral ketamine 3 mg/kg), or group K (oral ketamine 6 mg/kg). The allocation sequence was concealed from the investigator enrolling and assessing participants by using sequentially-numbered, opaque, sealed, and stapled envelopes.

Pre-anesthetic visits and communications with patients and their parents were conducted the day before surgery. Solid food was allowed until 8 h prior to the scheduled surgery time, and clear liquids were permitted until 3 h before this time. The investigator who prepared the concealed allocation envelopes was responsible for informing the nurse premedicating the children of the allocation group; this investigator was not involved with patient management or assessment during the trial. At 30 min before the induction of anesthesia, while the patient was still in the waiting room, a full-time nurse not involved in study measurements or evaluation administered intranasal dexmedetomidine, an oral ketamine syrup mixture (racemic ketamine and 5% glucose in a 1:2 ratio), or both to each patient, depending on the group assignment. Given that the concentration of the undiluted dexmedetomidine solution was 100 μg/ml, the volume administered with a 1-ml syringe was 0.02 ml/kg, which was sprayed evenly into both nostrils. The timing of premedication 30 min before induction was chosen based on the results of a previous clinical trial, which reported a sedation onset time of approximately 20–30 min [7].

The heart rate (HR) and pulse oxygen saturation (SpO_2_) were recorded before drug administration (T_0_) and 10 min (T_10_), 20 min (T_20_), and 30 min (T_30_) after drug administration. The pulse oximeter was decorated with cartoon stickers and the children were distracted with stuffed toys to minimize stress associated with this monitoring. Sedation scores were assessed according to the Sedation Scale (SS-5, Table 1) at T_10_, T_20_, and T_30_. The time of sedation onset, defined as the time from drug administration to the time when the SS-5 score reached three points, was also recorded. Separation state was assessed at 30 min after premedication and designated as satisfactory separation if the Emotional State Scale (ESS-4, Table 1) score was no more than two points.

**Table 1:** Sedation Scale (SS-5) and Emotional State Scale (ESS-4)

Sedation Score	
1	Rarely awake, needs shaking or shouting to wake up
2	Asleep, eyes closed, wake up when called softly or lightly touched
3	Sleepy, but eyes open spontaneously
4	Awake
5	Agitated
Emotional State Score	
1	Calm
2	Apprehensive, not smiling, tentative behavior, withdrawn
3	Crying
4	Thrashing, crying with movements of the arms and legs, resisting

A trained nurse anesthetist with at least 5 years clinical experience performed venous cannulation; this person was blinded to the patient’s group assignment. Successful venous cannulation was defined as an ESS-4 ≤ 2 at the time of attempted cannulation, regardless of whether the vein was actually cannulated on the first attempt. Children whose vein was cannulated were induced with intravenous propofol 3 mg/kg, fentanyl 3 μg/kg, and mivacurium chloride 0.2 mg/kg. Children for whom intravenous cannulation was not possible on the first attempt and those with an ESS-4 score ≥ 3 points were induced with sevoflurane.

After induction, an appropriately-sized laryngeal mask airway (LMA™, The Laryngeal Mask Company Limited, Singapore) was inserted. Ophthalmic 0.4% oxybuprocaine drops were instilled into the affected eye to provide topical anesthesia. Anesthesia was maintained with a combination of remifentanil 0.15 μg/kg/min and sevoflurane 1.0 minimum alveolar concentration. The attending anesthetists were unaware of the type of premedication administered. At the conclusion of the surgical procedure, the children were transferred to the recovery room with the LMA in place. The time of LMA removal (from the end of surgery to the time of removal) and the time of resumption of mental orientation (from the end of surgery to the time when the child opened his or her eyes and became oriented) were recorded. The children were discharged to the ward when their Aldrete score was at least 9. Follow-up evaluations were performed at 4, 8, 12, and 24 h after surgery, and adverse events were recorded by an observer who was blinded to the child’s group assignment and did not participate in patient care.

Based on preliminary experiments, the rate of success of venous cannulation with dexmedetomidine alone was approximately 47%. Prospective power analysis revealed that 39 patients per group offered a 90% likelihood of detecting a clinically relevant (30%) increase in the successful venous cannulation rate, with a type I error of 5%. Estimating a loss of 10–15% of subjects during the trial, the number of patients in each group was tentatively set at approximately 45.

The results were analyzed with SPSS 15.0 software (SPSS Inc., Chicago, IL, USA). Parametric data are presented as mean ± standard deviation, and one-way analysis of variance (ANOVA) was used to compare mean differences between groups for demographic data, time of sedation onset, time of LMA removal, and time of resumption of mental orientation. Two-way ANOVA, followed by post hoc tests, was used to analyze heart rates, considering treatment and time as two independent factors. Tukey-corrected post hoc test was used to adjust the observed significance level for the multiple comparisons performed. Chi-square test was used to compare qualitative data: rate of successful intravenous cannulation, type of diagnosis, and rate of postoperative adverse events. Wilcoxon’s rank sum test was to compare ranked data (sedation scores). *P* < 0.05 was considered statistically significant.

## Results

**Fig. 1:**
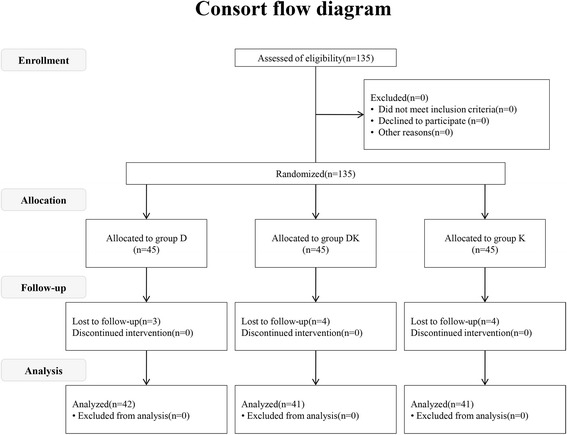
Consort flow diagram

Eligible participants were recruited in the study from April 2014 to June 2014. Eleven patients were excluded after randomization because of premedication failure (3, 4, and 4 patients in groups D, DK, and K, respectively). A total of 124 patients were included in the final statistical analyses (Fig. 1). Sex, age, weight, surgical diagnosis, duration of operation were similar among groups (Table 2).

**Table 2:** Demographic and clinical data. Values are presented as number or mean (standard deviation)

	Group D	Group DK	Group K	*P* Value	*F* Value	Effect Size (η^2^)
	*n* = 42	n = 41	n = 41			
Gender (male/female)	19/23	22/19	24/17	0.47	NA	NA
Age (years)	3.96 (1.50)	3.76 (1.36)	4.41 (1.28)	0.33	2.25	0.035
Weight (kg)	16.14 (4.47)	16.80 (3.20)	17.57 (3.23)	0.21	1.56	0.025
Diagnosis (cataract/ glaucoma/ strabismus)	9/ 13/ 20	10/ 8/ 23	11/ 10/ 20	0.79	NA	NA
Operation duration (min)	27.49 (5.13)	27.43 (5.11)	26.68 (5.17)	0.73	0.32	0.005

*NA* not applicable

The rate of successful venous cannulation was significantly higher in group DK and group K than in group D (*P* = 0.006), whereas the rate of satisfactory separation did not differ significantly among groups (*P* = 0.078) (Table 3). Sedation scores of the three groups showed no significant difference at T_10_; however, they were significantly lower in group D and group DK than in group K at T_20_ and T_30_ (*P* < 0.001) (Table 4). The time of sedation onset in group DK and group D was significantly shorter than in group K (*P* = 0.001). The time of LMA removal exhibited no significant difference among groups (*P* = 0.112). By contrast, the time of resumption of mental orientation was significantly different among groups (*P* = 0.001) (Table 3).

**Table 3:** Time of sedation onset, rate of satisfactory separation from parents, rate of successful intravenous cannulation, time of LMA removal, and time of resumption of mental orientation. Values are presented as mean (standard deviation) or number (proportion)

	Group D	Group DK	Group K	*P* Value	*F* Value	Effect Size (η^2^)
	*n* = 42	*n* = 41	*n* = 41			
Sedation onset (min)	20.62 (7.56)^*^	17.88 (6.61)^*^	25.20 (6.44)	<0.001	11.80	0.163
Satisfactory separation (%)	80.95	92.68	95.12	0.078	NA	NA
Successful intravenous cannulation (%)	47.62^**#**^	80.49	68.29	0.006	NA	NA
Removal of LMA (min)	15.60 (12.23)	12.02 (2.88)	14.63 (5.48)	0.111	2.24	0.035
Resumption of mental orientation (min)	73.83 (25.20)^* #^	90.29 (21.84)^*^	52.85 (16.89)	<0.001	30.91	0.338

*LMA* laryngeal mask airway, *NA* not applicable

^*^ *P* < 0.01, compared with group K; ^**#**^ *P* < 0.01, compared with group DK

**Table 4:** Sedation scores at 10, 20, and 30 min after premedication. Data are expressed as median (interquartile range)

	Group D	Group DK	Group K
	*n* = 42	n = 41	*n* = 41
10 min	3 (1)	3 (1)	4 (1)
20 min	1 (2) ^*^	1 (1) ^*^	3 (3)
30 min	1 (0.25) ^*^	1 (0) ^*^	3 (3)

^*^ *P* < 0.01, compared with group K

HR became gradually slower after premedication in group D and group DK; the HRs in group D and group DK were significantly lower than in group K at T_20_ and T_30_ (*P* = 0.005) (Table 5). SpO_2_ was stable and similar before transfer to the operating room in all groups. Five children treated with ketamine alone appeared to experience upper airway irritation during induction, which improved after intravenous injection of 1.0 mg/kg succinylcholine. No child in group D or group DK experienced respiratory complications during induction.

**Table 5:** Heart rates at different time points. Values are presented as mean (standard deviation)

	Group D	Group DK	Group K	*P* Value	*F* Value	Effect Size (η^2^)
	*n* = 42	*n* = 41	*n* = 41			
Baseline	111.60 (22.42)	109.61 (19.55)	106.53 (13.54)	0.472	0.75	0.012
10 min	100.98 (13.79)	102.39 (17.26)	104.00 (13.57)	0.656	0.42	0.007
20 min	93.90 (11.23)^*^	96.24 (12.74)^*^	105.22 (13.62)	<0.001	9.349	0.134
30 min	94.19 (14.89)^**#**^	92.85 (12.82)^*^	102.63 (13.29)	0.003	6.151	0.092

^*^ *P* < 0.01, compared with group K, ^#^ *P* < 0.05, compared with group K

The rates of vomiting, salivation, psychological/psychiatric adverse events, and respiratory adverse events were significantly increased in group K during the recovery period and the first 24 h after surgery. Hypoxia occurred in three patients because of irritation from secretions during removal of the LMA. In one patient, choking and breath-holding were observed, and the LMA was reinserted after deepening the level of anesthesia. Children in group D and group DK experienced fewer postoperative adverse events overall (*P* = 0.0152) than those in group K (Table 6).

**Table 6:** Postoperative adverse events

	Group D	Group DK	Group K
	*n* = 42	*n* = 41	*n* = 41
Vomiting	1^*^	3^#^	10
Psychological/ psychiatric events	0^*^	2^#^	8
Respiratory events	0^**#**^	0^#^	4

^*^ *P* < 0.01, compared with group K; ^**#**^ *P* < 0.05, compared with group K

## Discussion

Our study showed that a combination of intranasal dexmedetomidine 2 μg/kg and oral ketamine 3 mg/kg resulted in more successful venous cannulations and fewer perioperative adverse events compared with premedication using dexmedetomidine or ketamine alone. Dexmedetomidine produced satisfactory sedative effects, which were complemented by ketamine’s analgesic effects. Thus, 90% of children exhibited satisfactory sedation 30 min after premedication, and the rate of successful venous cannulation with the premedication combination was as high as 80%.

The aims of premedication for pediatric patients are to reduce preoperative separation anxiety and postoperative psychological trauma, to help the patient undergo smooth induction of anesthesia, and to ensure perioperative safety. Although intranasal dexmedetomidine was reported to produce acceptable sedative effects and improve the emotional state of children when separating from their parents [4, 7–9], dexmedetomidine as a sole agent has not been uniformly successful for invasive procedures [10]. To overcome some of the pitfalls of dexmedetomidine alone, recent literature has shown beneficial effects of combining dexmedetomidine and ketamine for procedural sedation in pediatric patients.

The superiority of combining dexmedetomidine and ketamine was fully demonstrated for awake fiberoptic nasotracheal intubation of adults [11] and for cardiac catheterization of children, when intravenous medication maintained the required depth of sedation [12, 13]. Intranasal instillation and oral administration are easy to perform, which may alleviate the fear of needles precipitated by intramuscular or intravenous injections and thereby reduce the occurrence of spasmodic sobbing, breath-holding, swallowing, abdominal distension, or other adverse events related to crying. Thus, administration of intranasal dexmedetomidine and oral ketamine was previously reported to be an optimal combination, allowing children to separate easily from their parents and accept intravenous cannulation, while maintaining hemodynamic stability and not causing excessive side effects or postoperative complications [14]. Likewise, a nebulized combination of low dose ketamine and dexmedetomidine was reported to produce more satisfactory sedation and a smoother induction of general anesthesia than nebulized ketamine or dexmedetomidine alone, with more rapid recovery and no significant side effects [15]. The current study also found that the combination of ketamine or dexmedetomidine significantly increased the success rate of venous cannulation, along with producing a smooth induction and stable recovery, without affecting the time of LMA removal. Although the time required for resumption of mental orientation was longer in patients receiving the combination, the children had stable vital signs and no adverse respiratory or cardiovascular events during the time before orientation returned.

Theoretically, a combination of dexmedetomidine and ketamine, which have complementary pharmacological characteristics, produce acceptable sedative and analgesic effects and can significantly accelerate the onset of sedation. The absolute bioavailability of dexmedetomidine has been reported to be approximately 82% following intranasal administration. The time of sedation onset for intranasal 1–4 μg/kg dexmedetomidine was approximately 15–45 min in healthy volunteers and children, with significant sedation observed for 1–2 h and an elimination half-life of 2.1–3.1 h [16]. Oral bioavailability of ketamine is poor (only 8–24%), and previous research indicates that children tend to be sedated within 20 min after oral ketamine. Pharmacodynamic analysis has shown that concentrations associated with arousal in children are analogous to those in adults, and the elimination half-life of oral ketamine is approximately 2–4 h [5].

Furthermore, the undesirable increase in airway secretions with ketamine administration is attenuated by the xerostomia induced by dexmedetomidine, while concurrent ketamine injection prevents bradycardia and hypotension reported with dexmedetomidine [6]. Side effects of delirium, nausea, and vomiting were observed after oral administration of 4–8 mg/kg ketamine for sedation [17]. Our study also showed that five children receiving ketamine alone had evidence of upper airway irritation and irregular breathing when inhalational mask induction was performed. Emergent intubation was required in some of these patients, and a higher incidence of postoperative nausea, vomiting, hypoxia, and other respiratory-related complications was noted with ketamine alone, which are clear drawbacks to the use of ketamine alone as premedication.

This study has some limitations. First, further stratification by age was not implemented, which possibly influenced our results, as Yuen et al. reported that higher doses of dexmedetomidine were required in younger children [7]. Secondly, children with an ESS-4 score ≥ 3 points or for whom venous cannulation was not possible on the first attempt underwent inhalational induction, which may have produced respiratory effects during the induction period that potentially affected the rate of respiratory-related adverse events after surgery.

## Conclusions

In summary, the combination of dexmedetomidine and ketamine produces definite sedative effects that help children separate quietly from their parents and smoothly accept induction of anesthesia, and is associated with fewer perioperative adverse events. This premedication combination is thus a reasonable option for use in clinical practice.

## Abbreviations

ANOVA: Analysis of variance
ESS: Emotional State Scale
HR: Heart rate
LMA: laryngeal mask airway
